# Supplementary material for: Epac1 increases myosin regulatory light-chain phosphorylation, energetic cost of contraction, and susceptibility to heart failure
Source: PLoS One. 2025 Jun 17;20(6):e0325986. doi: 10.1371/journal.pone.0325986 (PMC12173384; doi:10.1371/journal.pone.0325986)
Supplement: S1 Data — (PDF) [file pone.0325986.s001.pdf]

S1 Data

Epac1 increases myosin regulatory light-chain phosphorylation, energetic cost of contraction, and susceptibility to heart failure

Running title: Role of Epac1 in cardiac myofilament function

Yoshiki Ohnuki[1,2], Kenji Suita[1,2], Misao Ishikawa[3], Yasumasa Mototani[1], Megumi Nariyama[4], Aiko Ito[5], Ichiro Matsuo[1,2,6], Yoshio Hayakawa[1,7], Akinaka Morii[1,6], Takao Mitsubayashi[1], Yasutake Saeki[1], Yoshihiro Ishikawa[2], Satoshi Okumura[1,2]*

[1] Department of Physiology, Tsurumi University School of Dental Medicine, Yokohama, Japan

[2] Cardiovascular Research Institute, Yokohama City University Graduate School of Medicine, Yokohama, Japan

[3] Department of Oral Anatomy, Tsurumi University School of Dental Medicine, Yokohama Japan

[4] Department of Pediatric Dentistry, Tsurumi University School of Dental Medicine, Yokohama Japan

[5] Department of Orthodontics, Tsurumi University School of Dental Medicine, Yokohama, Japan

[6] Department of Periodontology, Tsurumi University School of Dental Medicine, Yokohama Japan

[7] Department of Dental Anesthesiology, Tsurumi University School of Dental Medicine, Yokohama, Japan

*Corresponding author: Satoshi Okumura: Department of Physiology, Tsurumi University School of Dental Medicine, Yokohama 230-8501, Japan; okumura-s@tsurumi-u.ac.jp; Tel. +81-(0)45-580-8476; Fax. +81-(0)45-585-2889.

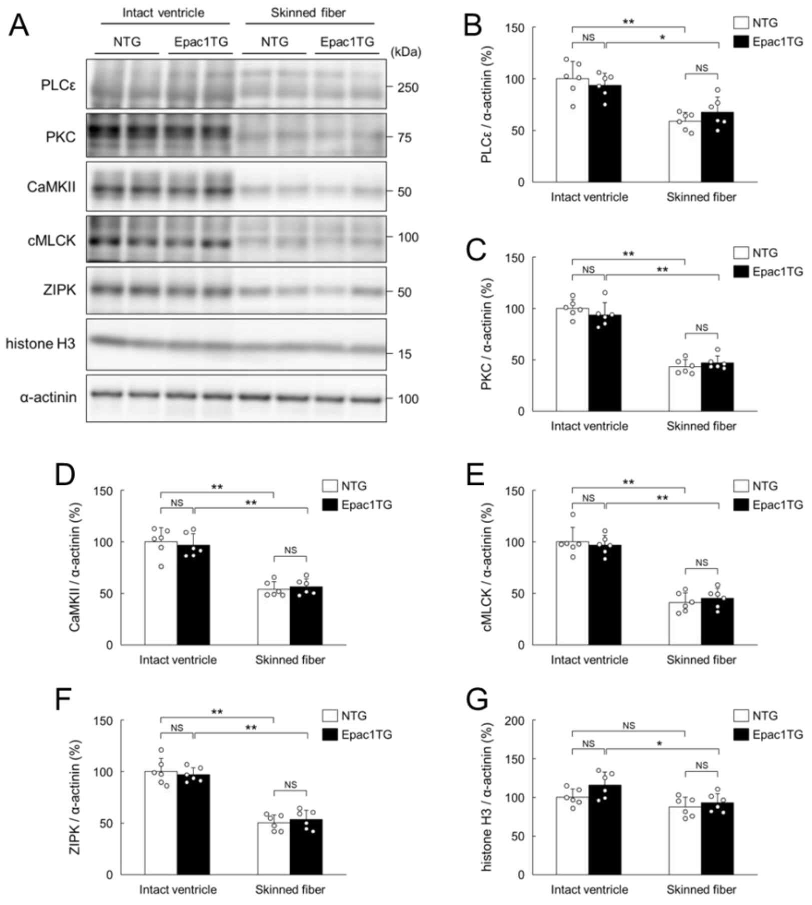

Supplementary Fig S1. **Expression of PLCε, PKC, CaMKII, cMLCK, ZIPK and histone H3 in the hearts of NTG and Epac1TG.**

(A) Representative western blotting of PLCε, PKC, CaMKII, cMLCK, ZIPK and histone H3 in total myocardium homogenate **(A; left)** and skinned myocardium **(A; right)** prepared from NTG and Epac1TG.

(B-G) The expression levels of PLCε **(B)**, PKC **(C)**, CaMKII **(D)**, cMLCK **(E)**, ZIPK **(F)** and histone H3 **(G)** were similar between NTG and Eapc1TG (P = NS by one-way ANOVA) not only in total myocardium homogenate but also in skinned myocardium. On the other hand, those in skinned myocardium were decreased to approximately to 40-80% of those in total myocardium homogenate ($^{*}P < 0.05$, $^{**}P < 0.01$ by one-way ANOVA) in both NTG and Epac1TG. The mean expression level in total myocardium homogenate prepared from NTG was taken as 100% in each case.

Bar blots represent means ± SD and open circles show individual data from biological replicates of NTG and Epac1TG (n = 6 each), each with 3 technical replicates.

PLCε, phospholipase Cε; PKC, protein kinase C; CaMKII, calcium/calmodulin-dependent protein kinase II; cMLCK, cardiac myosin light chain kinase; ZIPK, zipper interacting protein kinase.

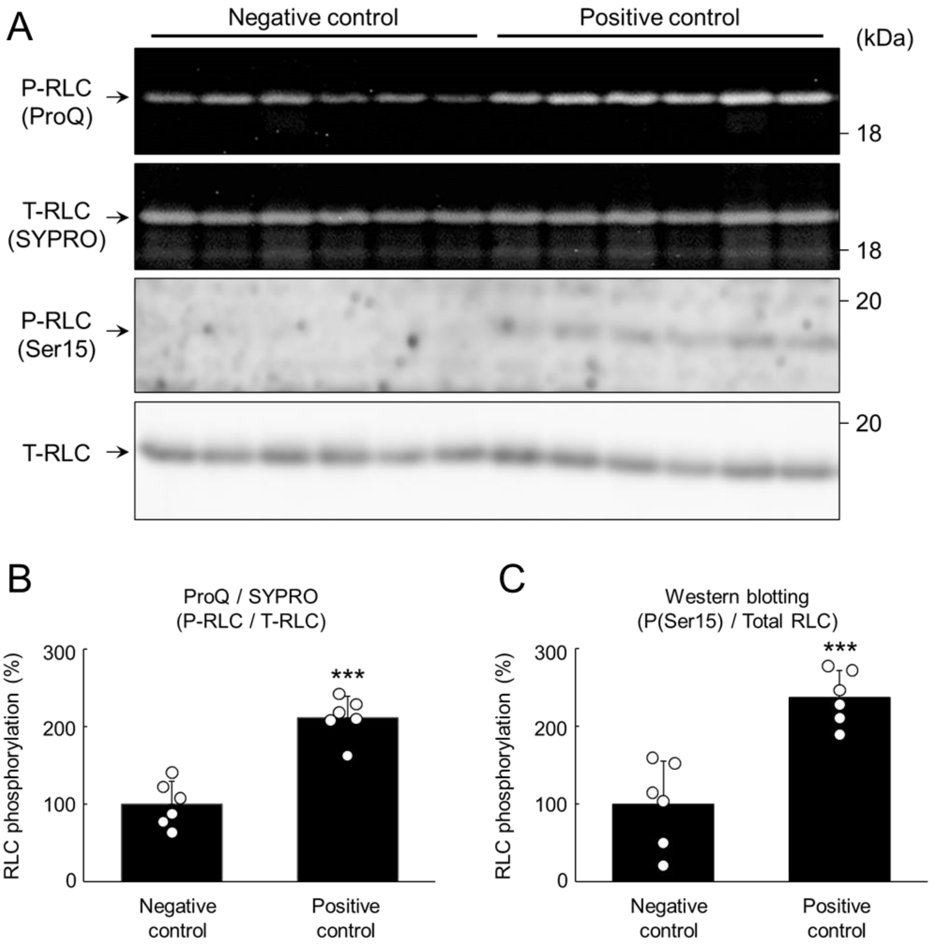

Supplementary Fig S2. Quantification of phosphorylation levels of RLC in skinned myocardium prepared from negative and positive controls.

(A) Representative ProQ/SYPRO staining **(Upper)** and western blotting **(lower)** of phosphorylated and total RLC in skinned myocardium prepared from hearts treated with (for negative control) or without (for positive control) Langendorff perfusion with a calcium-free Tyrode's solution for 1 hr.

(B, C) RLC phosphorylation in the positive control was significantly greater than that in the negative control ($^{***}P < 0.001$ by unpaired *t*-test) in both ProQ/SYPRO staining (100 ± 29 vs. 211 ± 27%) **(B)** and western blotting (100 ± 55 vs. 237 ± 35%) **(C)**. The magnitudes of the increase were similar between ProQ/SYPRO staining **(B)** and western blotting **(C)**. The mean phosphorylation level in the negative control was taken as 100% in each determination.

Bar blots represent means ± SD and open circles show individual data from biological replicates (*n* = 6 each), each with 3 technical replicates.

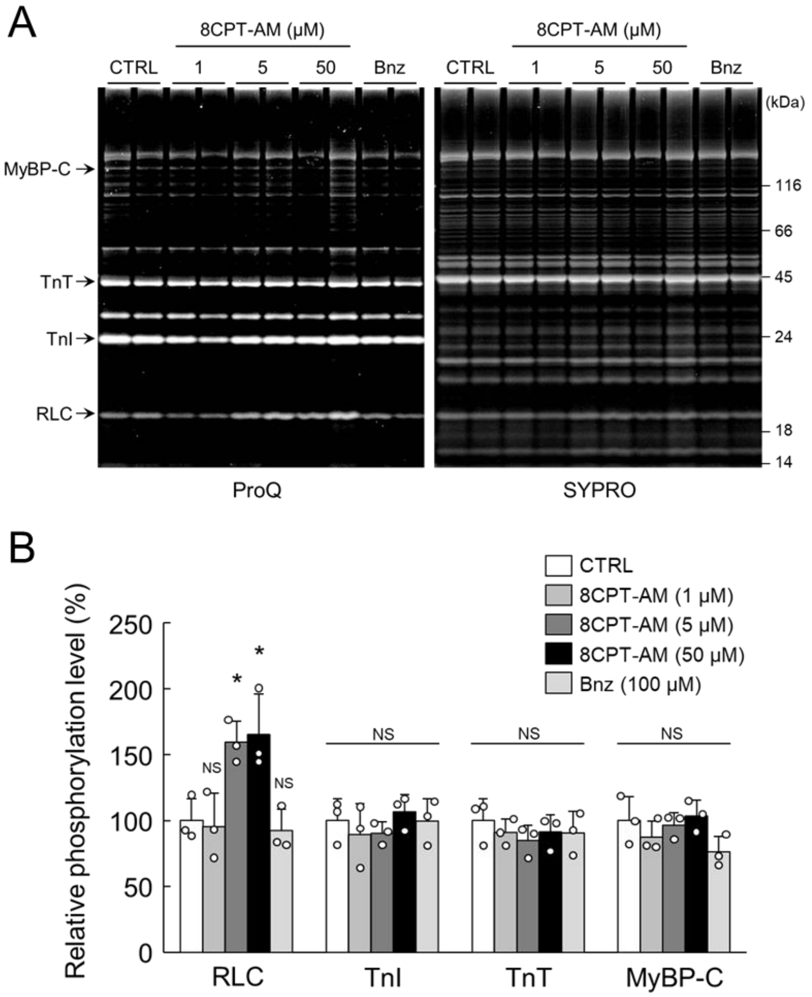

Supplementary Fig S3. RLC phosphorylation was increased in skinned myocardium treated with 8CPT-AM.

(A) Representative SDS-PAGE patterns of skinned myocardium treated with Epac activator, 8CPT-AM (1 µM, 5 µM, 50 µM for 30 min), or PKA activator, 6-Bnz-cAMP (Bnz; 100 µM for 30 min). The gel was stained with Pro-Q Diamond and subsequently stained with SYPRO Ruby. RLC, myosin regulatory light chain; TnI, troponin I; TnT, troponin T; MyBP-C, myosin binding protein-C.

(B) RLC phosphorylation in skinned myocardium treated with 8CPT-AM at 5 µM and 50 µM was significantly greater than in the control ($^{*}P < 0.05$ vs. Control by one-way ANOVA). However, phosphorylation levels of TnI, TnT and MyBP-C were similar among the five groups (P = NS by one-way ANOVA). The mean phosphorylation level in the control was taken as 100% in each case.

Bar blots represent means ± SD and open circles show individual data from biological replicates (n = 3 each), each with 3 technical replicates.

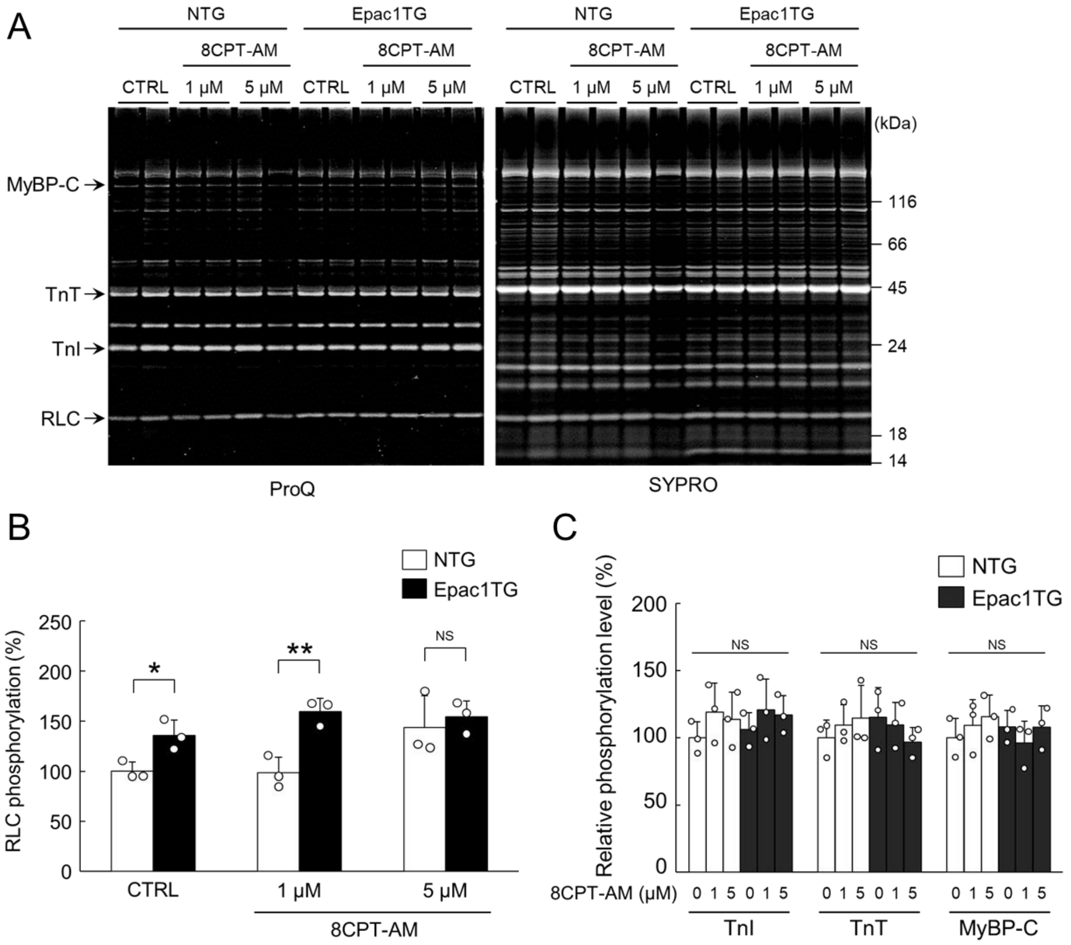

Supplementary Fig S4. Effects of 8CPT-AM on the phosphorylation status of RLC, TnI, TnT and MyBP-C in skinned myocardium prepared from NTG and Epac1TG.

(A) Representative SDS-PAGE patterns of skinned myocardium treated with or without Epac activator, 8CPT-AM (1 µM or 5 µM for 30 min). The gel was stained first with Pro-Q Diamond and then stained with SYPRO Ruby.

(B) RLC phosphorylation in Epac1TG was significantly greater than that in NTG without the treatment of 8CPT-AM treatment (Control) or after 8CPT-AM treatment at 1 µM ($^{*}P < 0.05$, $^{**}P < 0.01$ by unpaired *t*-test). However, RLC phosphorylation level was similar and might be saturated in both NTG and Epac1TG after 8CPT-AM treatment at 5 µM (*P* = NS by unpaired *t*-test). The mean phosphorylation level of RLC in the control NTG was taken as 100% in each determination.

(C) In contrast to the case of RLC, phosphorylation levels of TnI, TnT and MyBP-C were similar among the 6 groups (*P* = NS by one-way ANOVA). The mean phosphorylation level of NTG without 8CPT-AM treatment was taken as 100% in each case.

Bar blots represent means ± SD and open circles show individual data from biological replicates (*n* = 3 each), each with 3 technical replicates.

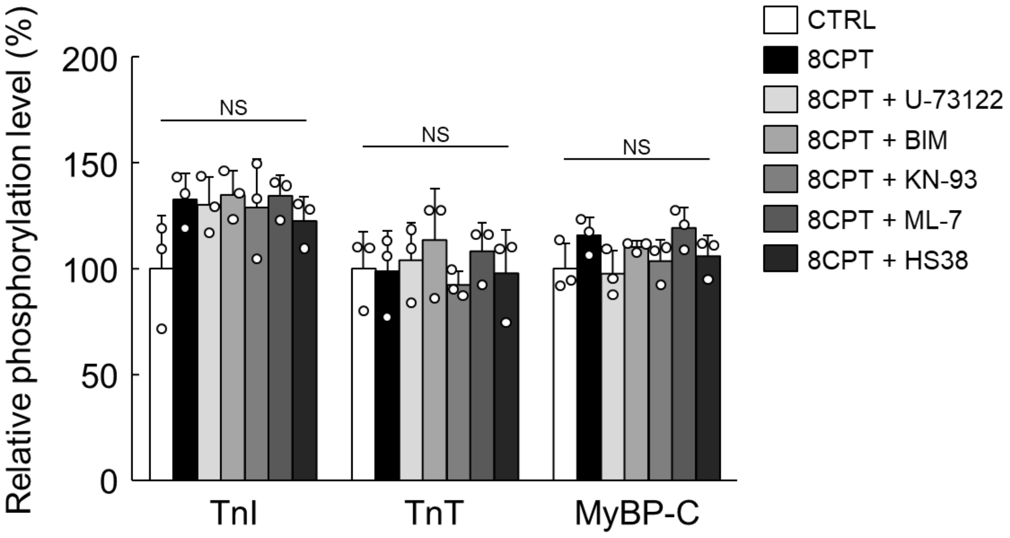

Supplementary Fig S5. Effects of 8CPT-AM on the phosphorylation status of TnI, TnT and MyBP-C in skinned myocardium prepared from WT mice.

As shown in Fig. 5, skinned myocardium was treated with 8CPT-AM (5 μM for 30 min) in the presence or absence of PLC inhibitor (U73122, 5 μM), PKC inhibitor (BIM, 1 μM), CaMKII inhibitor (KN-93, 2 μM), MLCK inhibitor (ML-7, 10 μM) or ZIPK inhibitor (HS38, 50 μM). In contrast to the case of RLC (Fig. 5B), phosphorylation levels of TnI, TnT and MyBP-C were similar among the 7 groups (*P* = NS by one-way ANOVA). The mean phosphorylation level in the control was taken as 100% in each determination.

Bar blots represent means ± SD and open circles show individual data from biological replicates (*n* = 3 each), each with 3 technical replicates.

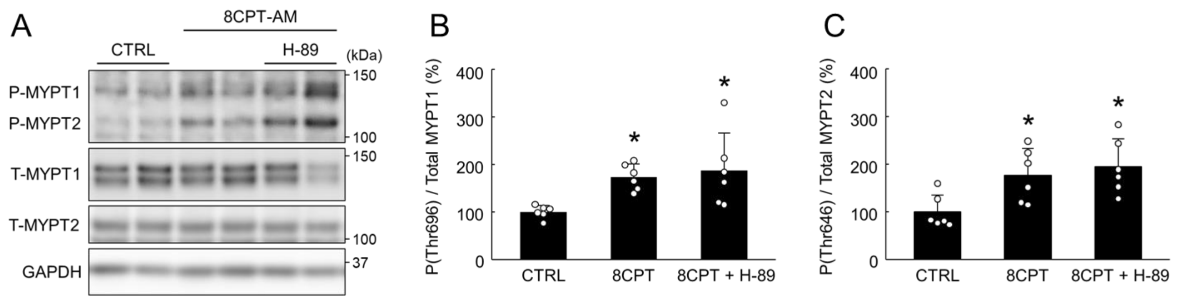

Supplementary Fig S6. Epac activation with 8CPT-AM induced MYPT phosphorylation in the perfused hearts.

(A) Representative western blots of phosphorylated (Thr 696) and total MYPT1 as well as phosphorylated (Thr 646) and total MYPT2 in total myocardium homogenate prepared from WT hearts perfused with oxygenated Tyrode's solution containing 8CPT-AM (2 µM) or 8CPT-AM (2 µM) plus PKA inhibitor, H-89 (5 µM), for 30 min.

(B, C) Phosphorylation levels of MYPT1 **(B)** and MYPT2 **(C)** were significantly increased by the treatment with 8CPT-AM ($^{*}P < 0.05$ vs. Control by one-way ANOVA) or 8CPT-AM plus H-89 ($^{*}P < 0.05$ vs. Control by one-way ANOVA), but the magnitudes of the increase were similar (P = NS by one-way ANOVA) for both MYPT1 and MYPT2. The mean phosphorylation level in the control was taken as 100% in each determination.

Bar blots represent means ± SD and open circles show individual data from biological replicates (n = 6 each), each with 3 technical replicates.

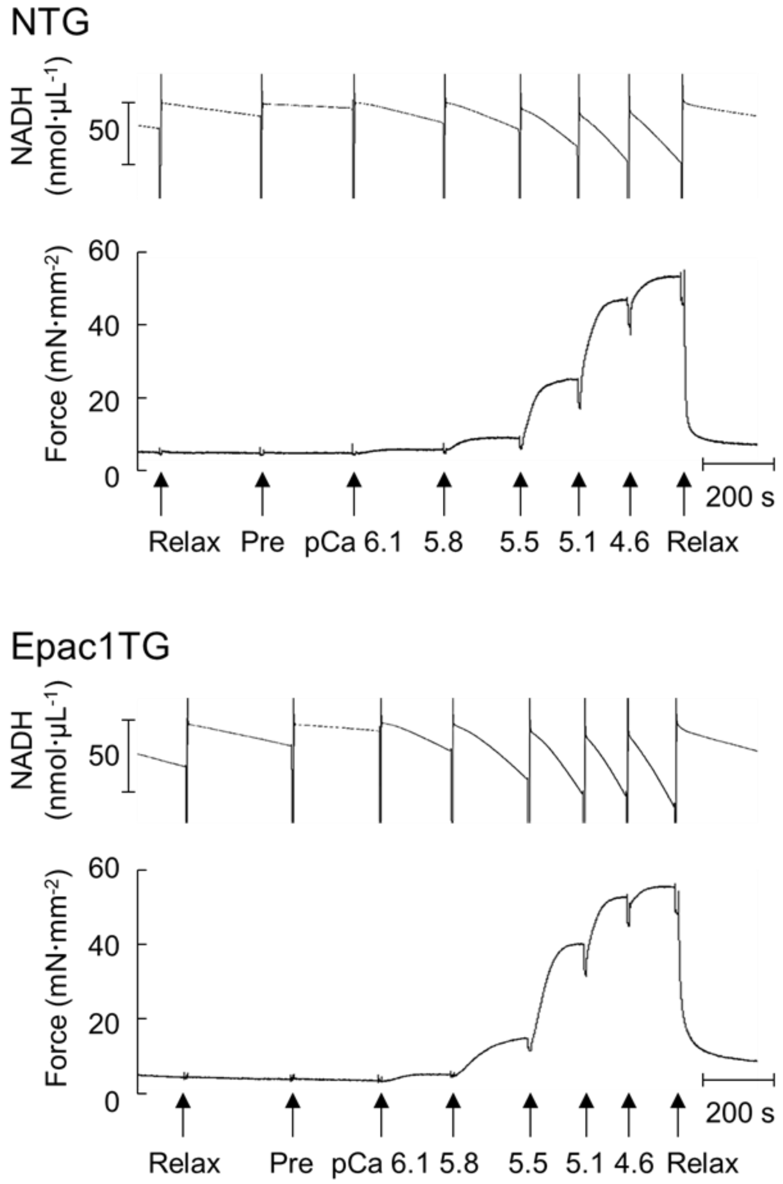

Supplementary Fig S7. **Simultaneous recording of Ca^{2+}-activated isometric force and NADH concentration in skinned myocardium prepared from Epac1TG.**
Simultaneous recordings of Ca^{2+}-activated isometric force (lower traces in each panel) and ATPase activity (upper traces in each panel) in skinned myocardium prepared from NTG **(upper)** and Epac1TG **(lower)**. ATPase activity (rate of ATP hydrolysis) was estimated from the slope representing the decrease rate of NADH concentration. The values of force and NADH consumption (i.e., ATPase activity) were normalized to the cross-sectional area and volume of skinned myocardium, respectively. To determine the pCa-isometric force and pCa-ATPase activity relationships, skinned preparations were sequentially bathed in solutions with pCa values ranging from 8.0 (Relax or Pre) to 4.6.

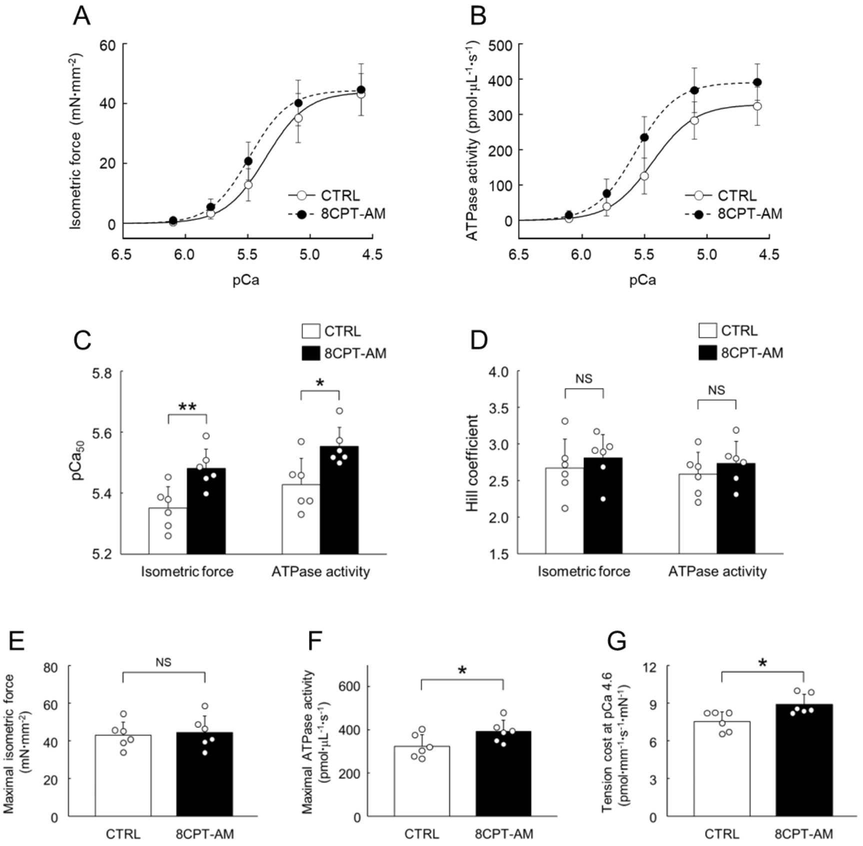

Supplementary Fig S8. Functional characterization of skinned myocardium treated with 8CPT-AM in WT.

(A, B) Average (mean ± SD) values of isometric force **(A)** and ATPase activity **(B)** at pCa 6.1, 5.8, 5.5, 5.1 and 4.6 were plotted for skinned myocardium with (closed circle) or without (open circle) 8CPT-AM treatment (5 μM for 30 min), and the data points were fitted to the Hill equation (solid line, Control [CTRL]; dashed line, 8CPT-AM-treated skinned myocardium, n = 10 each).

(C) Average pCa_{50} values (Ca^{2+} concentration required for half maximal effect) of isometric force **(left)** and ATPase activity **(right)** were significantly greater in 8CPT-AM than in CTRL (*$P < 0.05$, **$P < 0.01$ vs. CTRL by unpaired t-test).

(D) Average Hill coefficient of isometric force **(left)** and ATPase activity **(right)** were similar in CTRL and 8CPT-AM (P = NS by unpaired t-test).

(E) Maximal isometric force at pCa 4.6 was similar in CTRL and 8CPT-AM (P = NS by unpaired t-test).

(F-G) Maximal ATPase activity **(F)** and tension cost at pCa 4.6 **(G)** were significantly greater in 8CPT-AM than in CTRL (*$P < 0.05$ vs. CTRL by unpaired t-test).

Bar blots represent means ± SD and open circles show individual data from biological replicates (n = 6 each), each with 3 technical replicates.
