# Supplementary material for: Epac1 increases myosin regulatory light-chain phosphorylation, energetic cost of contraction, and susceptibility to heart failure
Source: PLoS One. 2025 Jun 17;20(6):e0325986. doi: 10.1371/journal.pone.0325986 (PMC12173384; doi:10.1371/journal.pone.0325986)

S2 Data

Epac1 increases myosin regulatory light-chain phosphorylation, energetic cost of contraction, and susceptibility to heart failure

Running title: Role of Epac1 in cardiac myofilament function

Yoshiki Ohnuki[1,2], Kenji Suita[1,2], Misao Ishikawa[3], Yasumasa Mototani[1], Megumi Nariyama[4], Aiko Ito[5], Ichiro Matsuo[1,2,6], Yoshio Hayakawa[1,7], Akinaka Morii[1,6], Takao Mitsubayashi[1], Yasutake Saeki[1], Yoshihiro Ishikawa[2], Satoshi Okumura[1,2]*

[1] Department of Physiology, Tsurumi University School of Dental Medicine, Yokohama, Japan

[2] Cardiovascular Research Institute, Yokohama City University Graduate School of Medicine, Yokohama, Japan

[3] Department of Oral Anatomy, Tsurumi University School of Dental Medicine, Yokohama Japan

[4] Department of Pediatric Dentistry, Tsurumi University School of Dental Medicine, Yokohama Japan

[5] Department of Orthodontics, Tsurumi University School of Dental Medicine, Yokohama, Japan

[6] Department of Periodontology, Tsurumi University School of Dental Medicine, Yokohama Japan

[7] Department of Dental Anesthesiology, Tsurumi University School of Dental Medicine, Yokohama, Japan

*Corresponding author: Satoshi Okumura: Department of Physiology, Tsurumi University School of Dental Medicine, Yokohama 230-8501, Japan; okumura-s@tsurumi-u.ac.jp; Tel. +81-(0)45-580-8476; Fax. +81-(0)45-585-2889.

Uncropped western blots used for Fig 1A.

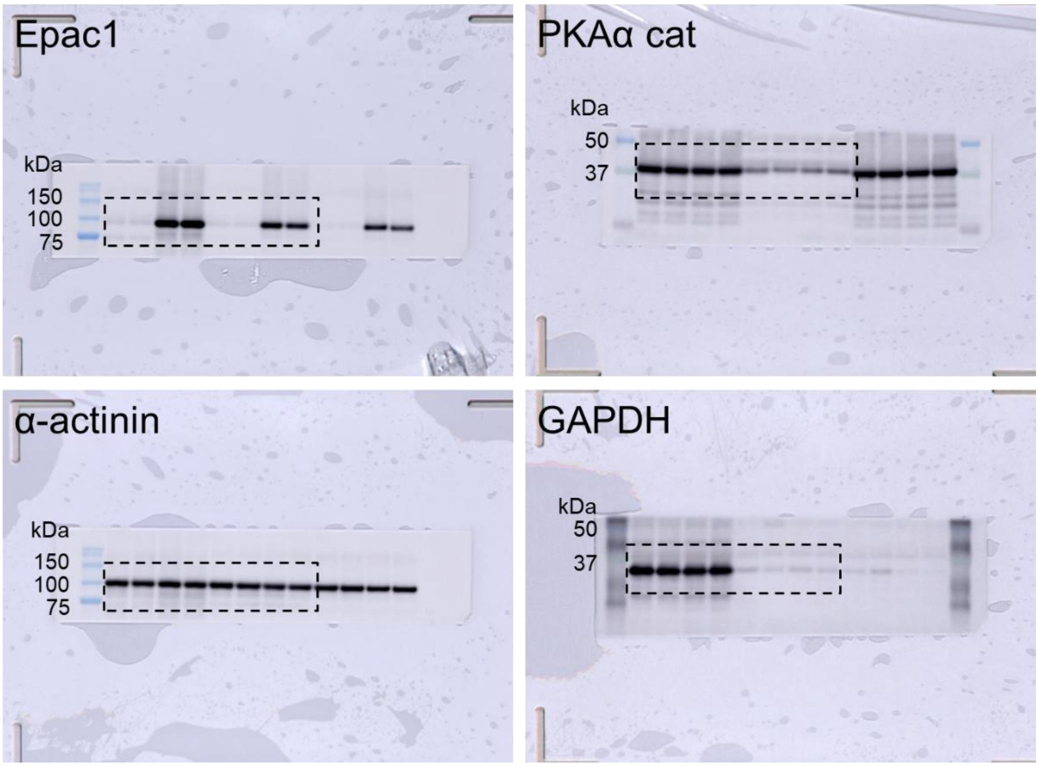

Uncropped silver-stained gel used for Fig 1D.

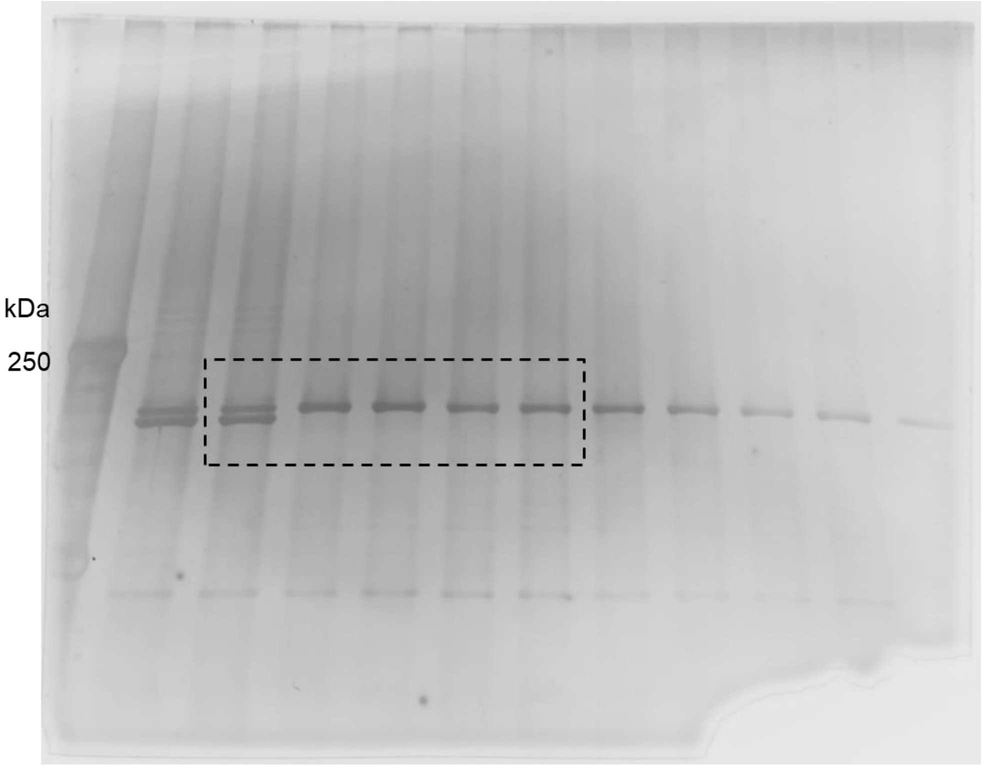

Uncropped ProQ-stained and SYPRO-stained gels used for Fig 2A.

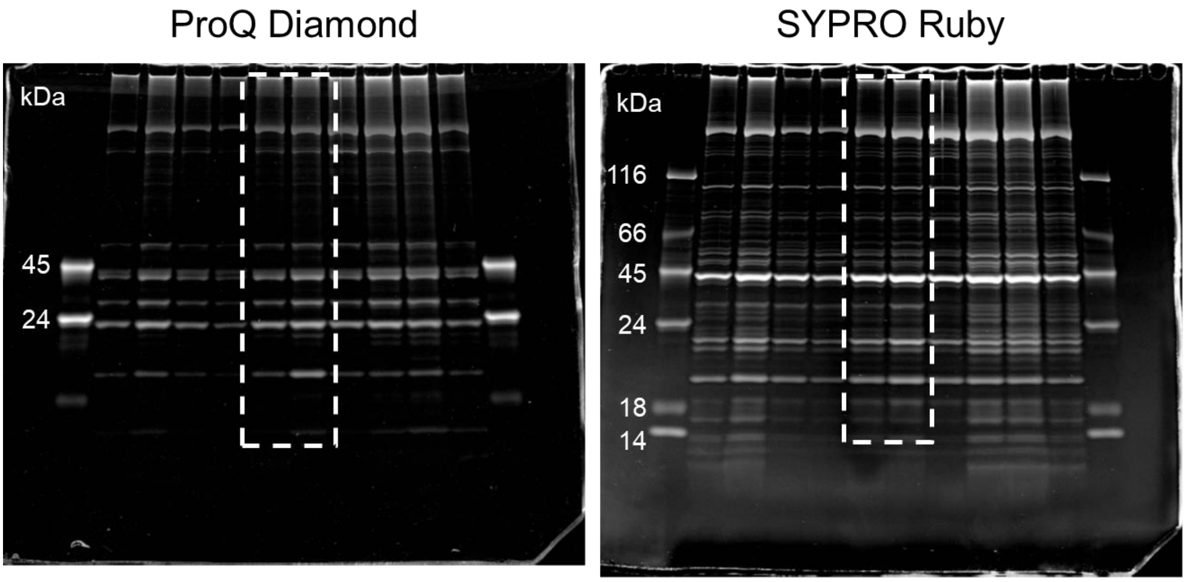

Uncropped ProQ-stained and SYPRO-stained gels used for Fig 3A.

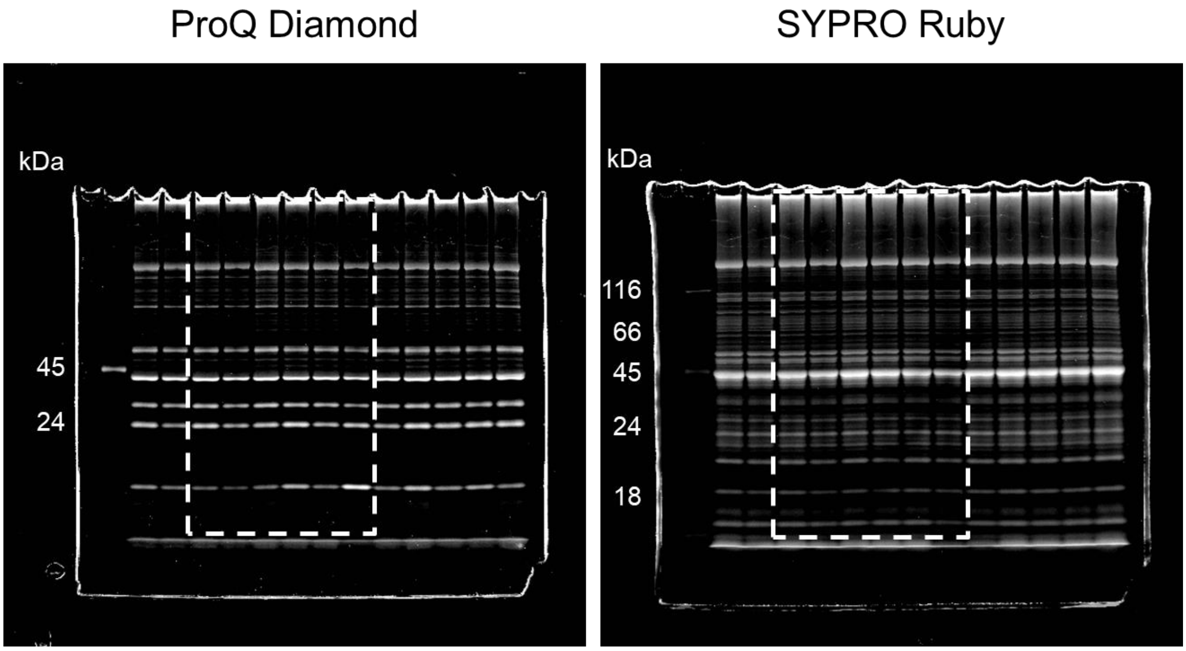

Uncropped ProQ-stained and SYPRO-stained gels used for Fig 4A.

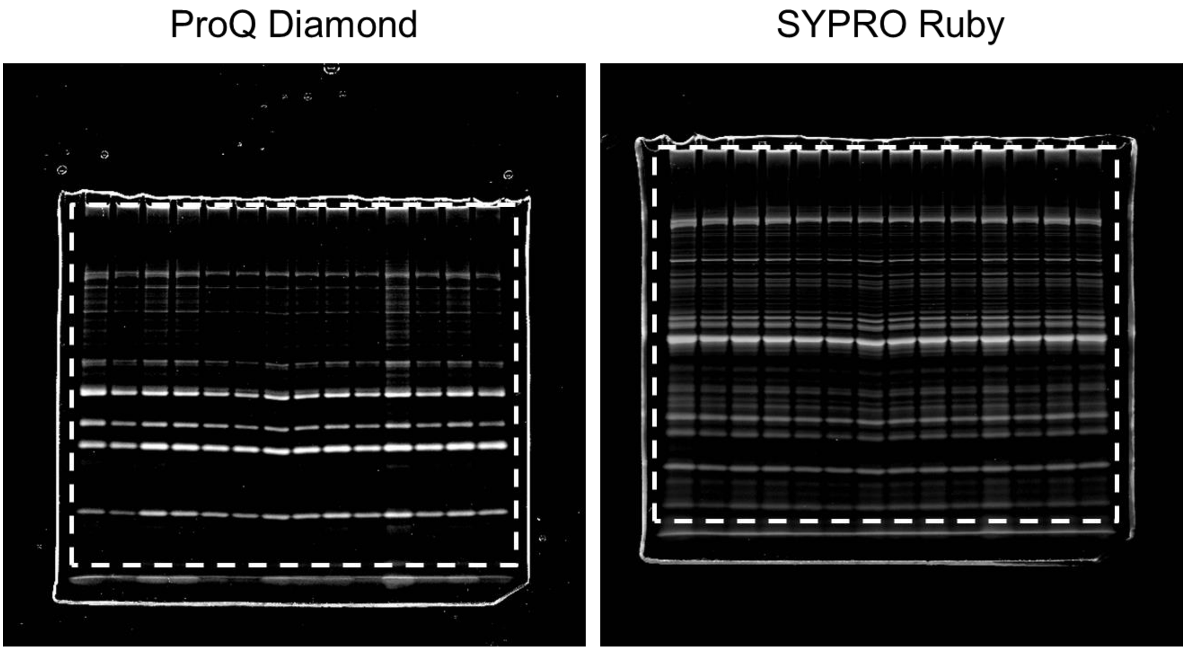

Uncropped western blots used for Fig 5A.

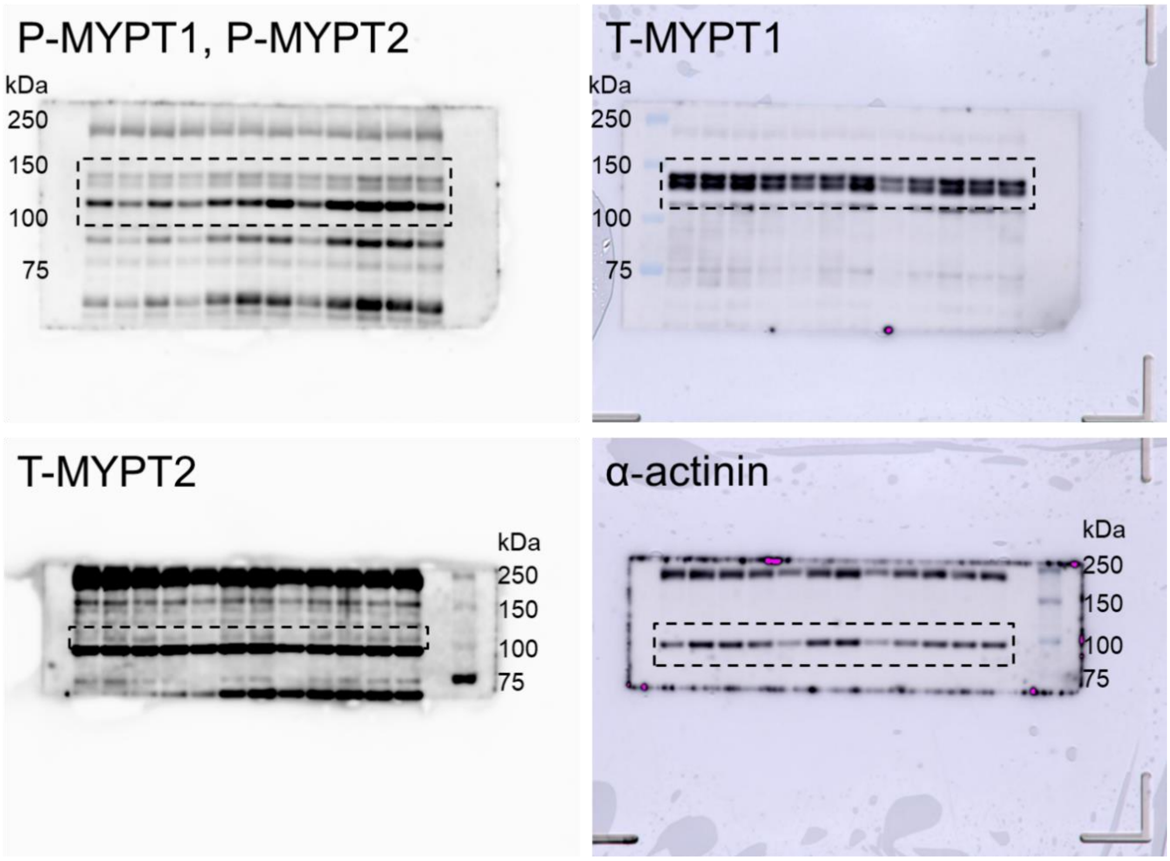

Uncropped western blots used for Fig 5D.

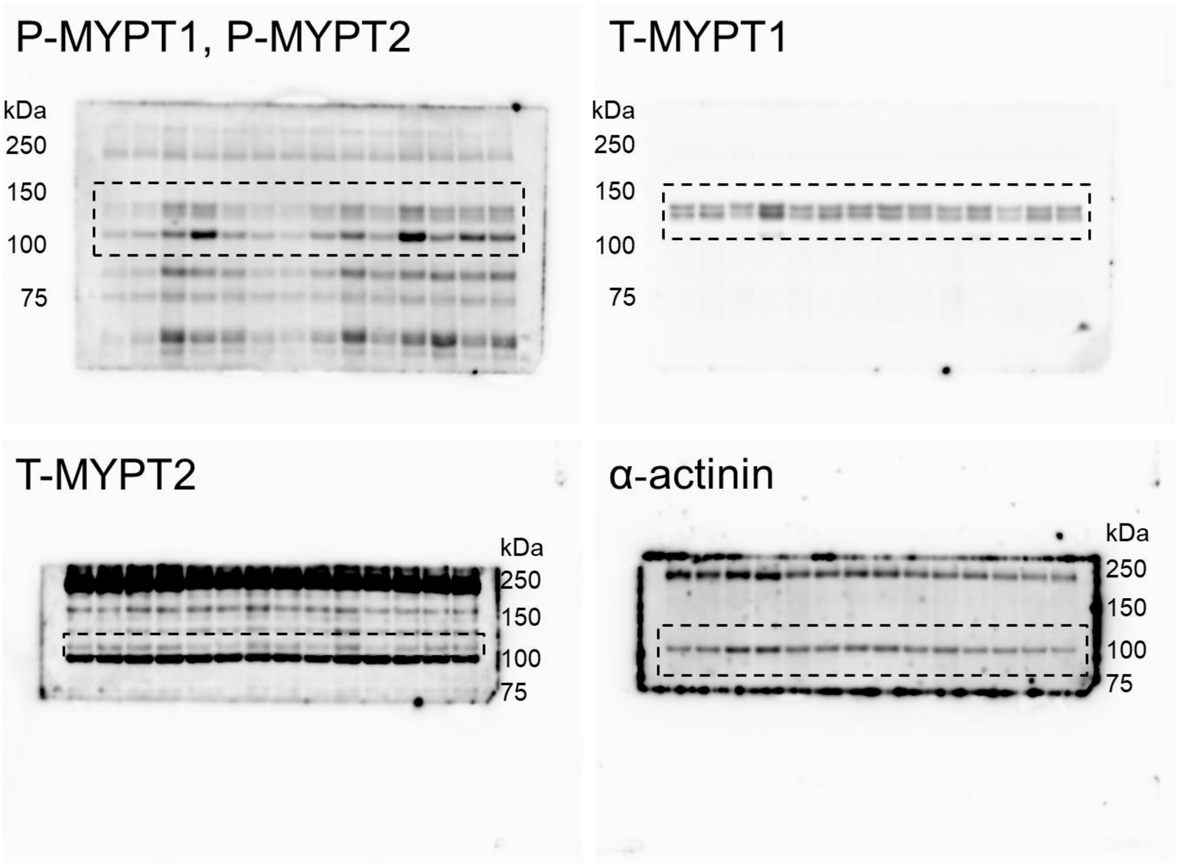

Uncropped western blots used for S1A Fig of S1 Data.

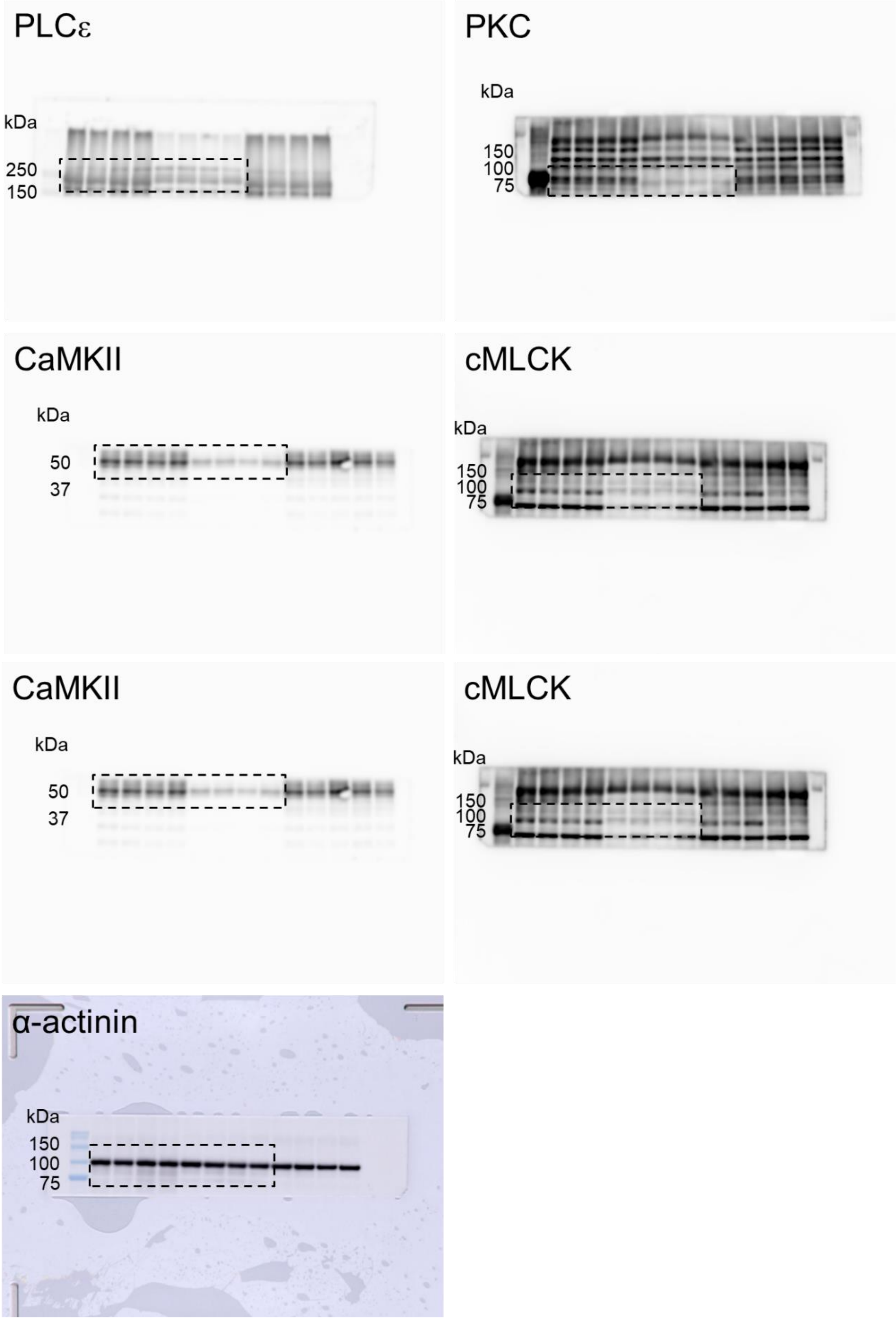

Uncropped ProQ/SYPRO-stained gels and western blots used for S2A Fig of S1 Data.

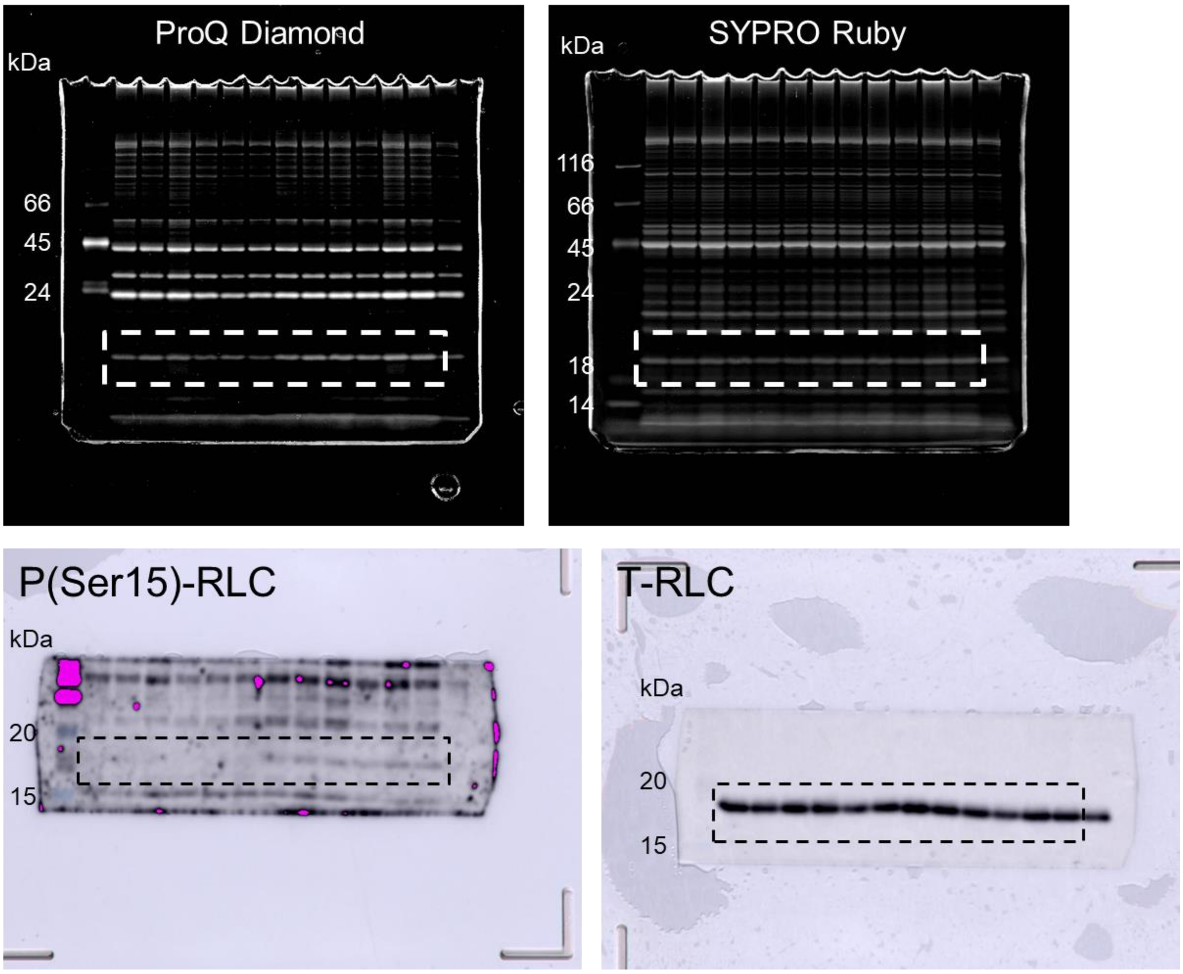

Uncropped ProQ-stained and SYPRO-stained gels used for S3A Fig of S1 Data.

ProQ Diamond

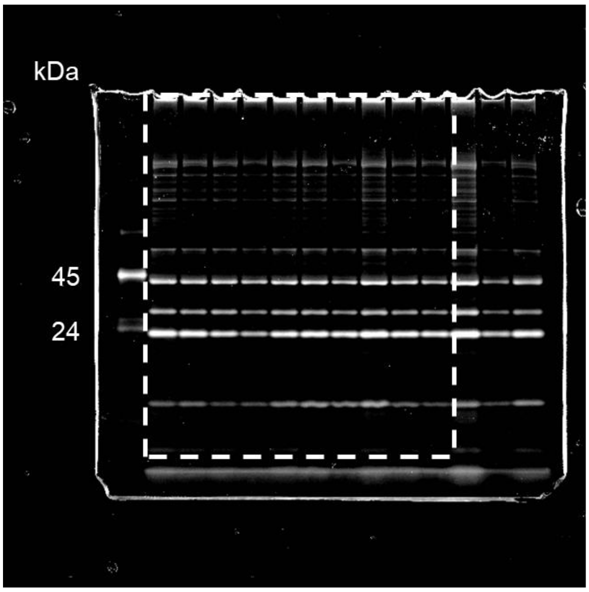

SYPRO Ruby

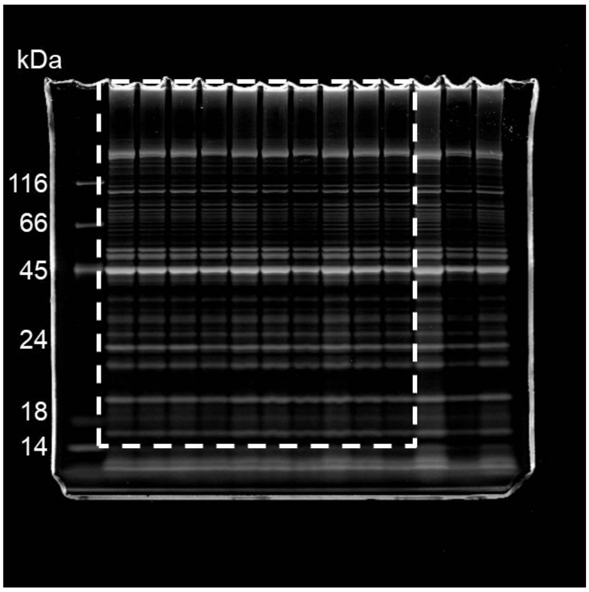

Uncropped ProQ-stained and SYPRO-stained gels used for S4A Fig of S1 Data.

ProQ Diamond

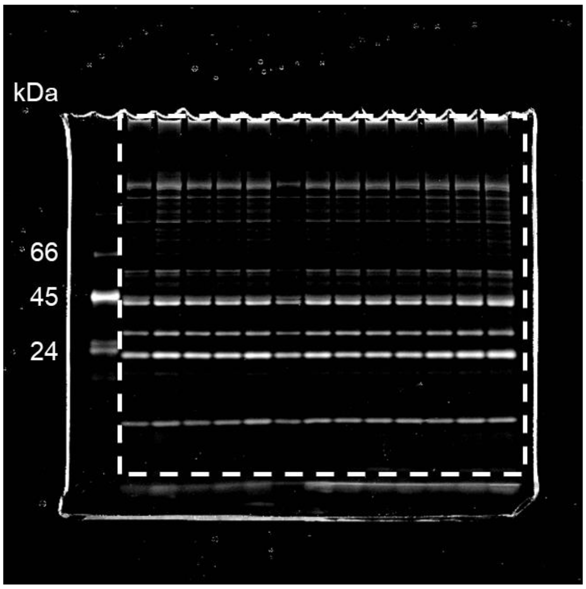

SYPRO Ruby

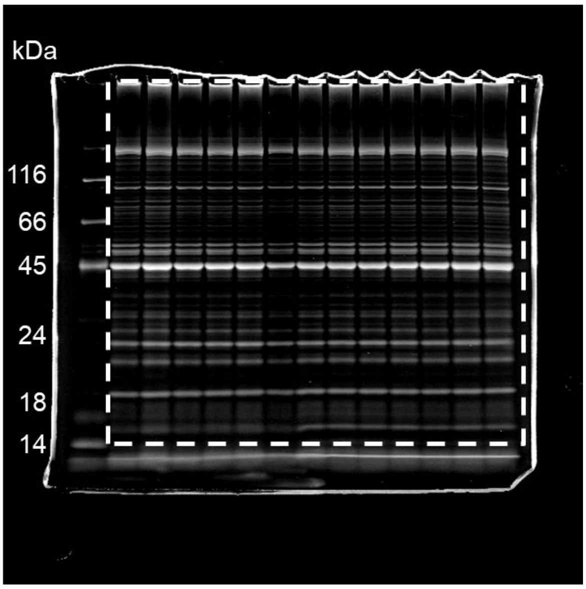

Uncropped western blots used for S6A Fig of S1 Data.

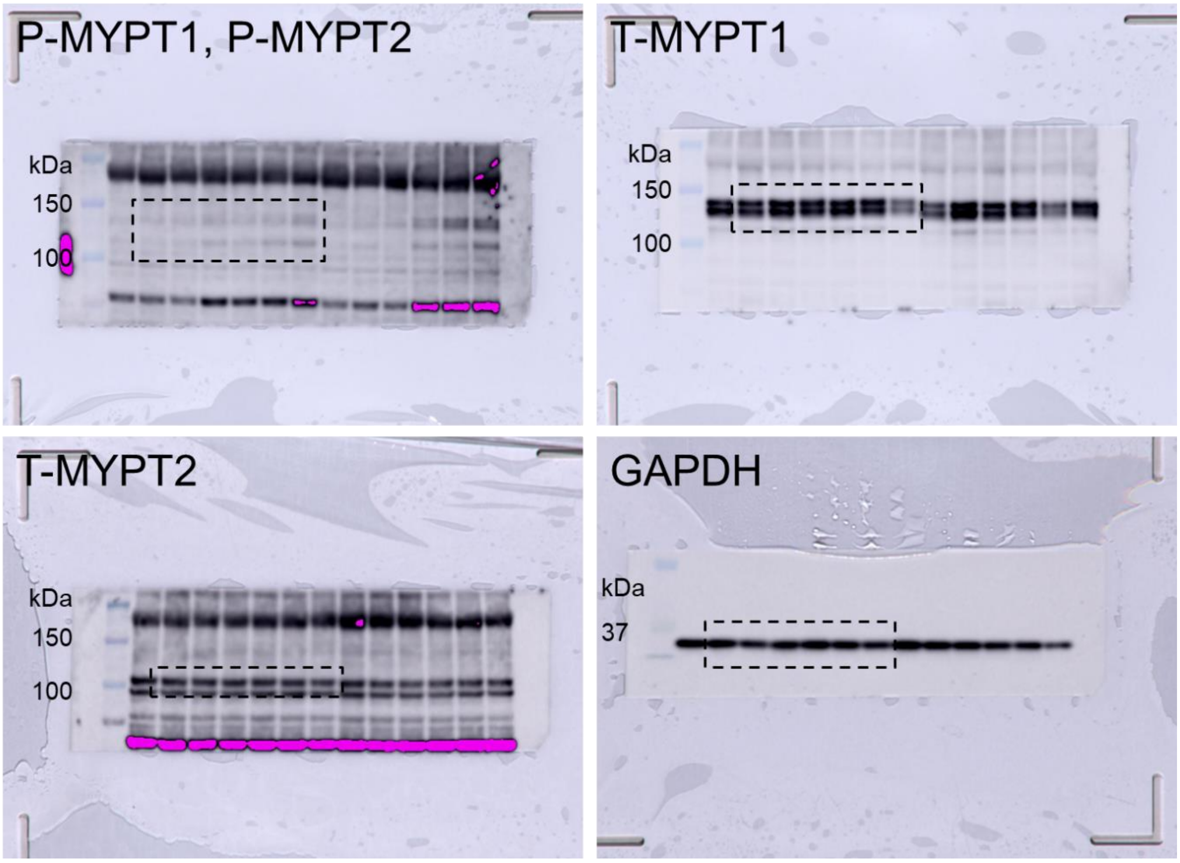

Supplement: S2 Data — (PDF) [file pone.0325986.s002.pdf]
