# Supplementary material for: Epac1 increases myosin regulatory light-chain phosphorylation, energetic cost of contraction, and susceptibility to heart failure
Source: PLoS One. 2025 Jun 17;20(6):e0325986. doi: 10.1371/journal.pone.0325986 (PMC12173384; doi:10.1371/journal.pone.0325986)
Supplement: Attachment — Submitted filename: Reviewer comments 2nd revise-08-add RS correction-01.pdf [file pone.0325986.s005.pdf]

Reviewer #1:

The authors have satisfactorily addressed my concerns with the additional points in the discussion.

Response:

Thank you.

Reviewer#2:

The Authors have done a good job of addressing the reviewer responses. However, their number of biological replicates and number of technical replicates per biological replicates are still confusing. That is very concerning.

At a minimum, the authors need to have 4-5 biological replicates (heart) for every experiment, and the experiments need to include all hearts among all the experiments This is not clear. Then, within each biological replicate there needs to be at least 3 technical replicates per each heart—for every experiment or assay. It is clear there are some measurements that meet this guideline, but as written many of them do not. Thus, it is not enough information to statistically and experimentally represent the observations for the study design. AS presented, it is a study design that is critically flawed and should not be published.

Response:

Thank you for your important comments. We apologize for the insufficient clarity regarding replicates, but should like to emphasize that the numbers of biological and technical replicates do in fact meet the necessary criteria. We have now clarified this in the text and figure legends as follows.

The numbers *n* of open circles in Fig 2, Fig 4, Fig 5, Fig 6, Fig S3, Fig S4, Fig S5 and Fig S8 in the original manuscript were larger than the numbers of biological replicates because we prepared two or more skinned fibers from one heart in some of the mice and the data from each skinned fiber from one heart were used for the analysis. We have revised the figures so that the number of open circles now corresponds to the number of biological replicates, each with 3 technical replicates that were averaged for further analysis.

We incorporated following sentences in the method sections of the revised manuscript (Page 13, Lines 1-3).

Open circles in the bar charts show individual data from biological replicates, each with 3 technical replicates that were averaged for subsequent analysis.

We also incorporated the following sentences in the figure legends of the revised manuscript.

in legends of Fig 1 (Page 48, Lines 15-16)
---Bar plots represent means ± SD and open circles show individual data from biological replicates of NTG and Epac1TG (*n* = 6 each), each with 3 technical replicates.

in legends of Fig 2 (Page 49, Lines 5-6)
---Bar plots represent means ± SD and open circles show individual data from biological replicates of NTG and Epac1TG (*n* = 4 each), each with 3 technical replicates.

in legends of Fig 3 (Page 50, Lines 1-3)
---Bar plots represent means ± SD and open circles show individual data from biological replicates of Control, 8CPT-AM, 8CPT-AM + H89 (*n* = 6 each), each with 3 technical replicates.

in legends of Fig 4 (Page 50, Lines 21-22)
---Bar plots represent means ± SD and open circles show individual data from biological replicates (*n* = 3 each), each with 3 technical replicates.

in legends of Fig 5 (Page 51, Lines 23-24)
---Bar plots represent means ± SD and open circles show individual data from biological replicates (*n* = 3 each), each with 3 technical replicates.

in legends of Fig 6 (Page 52, Lines 14-15)
---Bar plots represent means ± SD and open circles show individual data from biological replicates of NTG and Epac1TG (*n* = 4 each), each with 3 technical replicates.

in legends of Fig 7 (Page 53, Lines 12-13)

---Bar plots represent means ± SD and open circles show individual data from biological replicates of NTG and Epac1TG (*n* = 5 each), each with 3 technical replicates.

in legends of Fig S1 (S1 Data)

---Bar plots represent means ± SD and open circles show individual data from biological replicates of NTG and Epac1TG (*n* = 6 each), each with 3 technical replicates.

in legends of Fig S2 (S1 Data)

---Bar plots represent means ± SD and open circles show individual data from biological replicates (*n* = 6 each), each with 3 technical replicates.

in legends of Fig S3 (S1 Data)

---Bar plots represent means ± SD and open circles show individual data from biological replicates (*n* = 3 each), each with 3 technical replicates.

in legends of Fig S4 (S1 data)

---Bar plots represent means ± SD and open circles show individual data from biological replicates (*n* = 3 each), each with 3 technical replicates.

.

in legends of Fig S5 (S1 Data)

---Bar plots represent means ± SD and open circles show individual data from biological replicates (*n* = 3 each), each with 3 technical replicates.

in legends of Fig S6 (S1 Data)

---Bar plots represent means ± SD and open circles show individual data from biological replicates (*n* = 6 each), each with 3 technical replicates.

in legends of Fig S8 (S1 Data)

---Bar plots represent means ± SD and open circles show individual data from biological replicates (*n* = 6 each), each with 3 technical replicates.
